# 4-(Dimethyl­amino)pyridinium tribromide: whole mol­ecule disorder of cation and anion

**DOI:** 10.1107/S1600536809017048

**Published:** 2009-05-14

**Authors:** Seik Weng Ng

**Affiliations:** a Department of Chemistry, University of Malaya, 50603 Kuala Lumpur, Malaysia

## Abstract

In the title salt, C_7_H_11_N_2_^+^·Br_3_^−^, the cation and the near-linear anion [Br—Br—Br = 179.41 (8)°] both show whole-mol­ecule disorder about crystallographic twofold rotation axes. The cation is weakly hydrogen-bonded to the anion by an N—H⋯Br inter­action. The crystal studied was found to be a racemic twin, with a twin component of nearly 50%.

## Related literature

The compound is known commercially as 4-(dimethyl­amino)pyridine hydro­bromide perbromide, [C_7_H_10_N_2_]·[HBr]·[Br_2_]. The 4-dimethyl­amino­pyridinium cation furnishes a number of salts with organic and inorganic acids. For 4-dimethyl­amino­pyridinium bromide, see: Mayr-Stein & Bolte (2000 ▶). For dimethyl­amino­pyridinium chloride and its dihydrate, see: Bryant & King (1992 ▶); Chao *et al.* (1977 ▶).
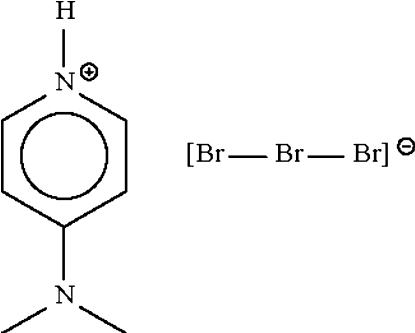


## Experimental

### 

#### Crystal data

C_7_H_11_N_2_^+^·Br_3_^−^
*M* *_r_* = 362.91
Orthorhombic, 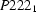

*a* = 4.1688 (1) Å
*b* = 8.8349 (2) Å
*c* = 14.7255 (4) Å
*V* = 542.35 (2) Å^3^
*Z* = 2
Mo *K*α radiation
μ = 11.11 mm^−1^
*T* = 100 K
0.20 × 0.15 × 0.10 mm

#### Data collection

Bruker SMART APEX CCD diffractometer
Absorption correction: multi-scan (*SADABS*; Sheldrick, 1996 ▶) *T*_min_ = 0.656, *T*_max_ = 1.000 (expected range = 0.216–0.329)
5156 measured reflections
1256 independent reflections
1114 reflections with *I* > 2σ(*I*)
*R*_int_ = 0.025

#### Refinement

*R*[*F* ^2^ > 2σ(*F* ^2^)] = 0.021
*wR*(*F* ^2^) = 0.051
*S* = 0.98
1256 reflections
100 parameters
60 restraints
H-atom parameters constrained
Δρ_max_ = 0.42 e Å^−3^
Δρ_min_ = −0.34 e Å^−3^
Absolute structure: Flack (1983 ▶), 480 Friedel pairs
Flack parameter: 0.47 (4)

### 

Data collection: *APEX2* (Bruker, 2008 ▶); cell refinement: *SAINT* (Bruker, 2008 ▶); data reduction: *SAINT*; program(s) used to solve structure: *SHELXS97* (Sheldrick, 2008 ▶); program(s) used to refine structure: *SHELXL97* (Sheldrick, 2008 ▶); molecular graphics: *X-SEED* (Barbour, 2001 ▶); software used to prepare material for publication: *publCIF* (Westrip, 2009 ▶).

## Supplementary Material

Crystal structure: contains datablocks global, I. DOI: 10.1107/S1600536809017048/hb2966sup1.cif

Structure factors: contains datablocks I. DOI: 10.1107/S1600536809017048/hb2966Isup2.hkl

Additional supplementary materials:  crystallographic information; 3D view; checkCIF report

## Figures and Tables

**Table 1:** Hydrogen-bond geometry (Å, °)

*D*—H⋯*A*	*D*—H	H⋯*A*	*D*⋯*A*	*D*—H⋯*A*
N1—H1⋯Br2	0.88	2.42	3.286 (2)	167

**Fig. 1.**
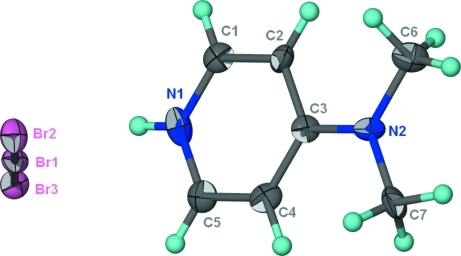
Thermal ellipsoid plot (Barbour, 2001) of [C7H11N2][Br3] at the 70% probability level. Hydrogen atoms are drawn as spheres of arbitrary radius.

## supplementary crystallographic information

### Experimental

Commercially-available 4-dimethylaminopyridine hydrobromide perbromide was recrystallized from ethanol to give colourless blocks of (I).

### Refinement

The Br_3_ anion lies on a twofold rotation axis, but it was allowed to refine off this symmetry element as a three-atom species.

The cation is disordered about another twofold rotation axis; this was refined as a cation with its atoms of half occupancies. The pyridyl portion was refined as a rigid hexagon of 1.39 Å sides; the pair of N–C_methyl_ distances were restrained to within 0.01 Å of each other. The cation was restrained to be nearly planar, and the anisotropic displacement factors were restrained to be nearly isotropic.

The hydrogen atoms were placed at calculated positions (C–H 0.95, N–H 0.88 Å) and refined as riding with *U*_iso_(H) = 1.2*U*_eq_(C,*N*).

### Crystal data

**Table:**

C_7_H_11_N_2_^+^·Br_3_^−^	*F*(000) = 344
*M**_r_* = 362.91	*D*_x_ = 2.222 Mg m^−^^3^
Orthorhombic, *P*222_1_	Mo *K*α radiation, λ = 0.71073 Å
Hall symbol: P 2c 2	Cell parameters from 2094 reflections
*a* = 4.1688 (1) Å	θ = 2.7–28.3°
*b* = 8.8349 (2) Å	µ = 11.11 mm^−^^1^
*c* = 14.7255 (4) Å	*T* = 100 K
*V* = 542.35 (2) Å^3^	Block, colorless
*Z* = 2	0.20 × 0.15 × 0.10 mm

### Data collection

**Table:**

Bruker SMART APEX CCD diffractometer	1256 independent reflections
Radiation source: fine-focus sealed tube	1114 reflections with *I* > 2σ(*I*)
graphite	*R*_int_ = 0.025
ω scans	θ_max_ = 27.5°, θ_min_ = 2.3°
Absorption correction: multi-scan (*SADABS*; Sheldrick, 1996)	*h* = −5→5
*T*_min_ = 0.656, *T*_max_ = 1.000	*k* = −11→11
5156 measured reflections	*l* = −19→19

### Refinement

**Table:**

Refinement on *F*^2^	Secondary atom site location: difference Fourier map
Least-squares matrix: full	Hydrogen site location: inferred from neighbouring sites
*R*[*F*^2^ > 2σ(*F*^2^)] = 0.021	H-atom parameters constrained
*wR*(*F*^2^) = 0.051	*w* = 1/[σ^2^(*F*_o_^2^) + (0.0322*P*)^2^] where *P* = (*F*_o_^2^ + 2*F*_c_^2^)/3
*S* = 0.98	(Δ/σ)_max_ = 0.001
1256 reflections	Δρ_max_ = 0.42 e Å^−^^3^
100 parameters	Δρ_min_ = −0.33 e Å^−^^3^
60 restraints	Absolute structure: Flack (1983), 480 Friedel pairs
Primary atom site location: structure-invariant direct methods	Flack parameter: 0.47 (4)

### Special details

**Table:**

Geometry. All esds (except the esd in the dihedral angle between two l.s. planes) are estimated using the full covariance matrix. The cell esds are taken into account individually in the estimation of esds in distances, angles and torsion angles; correlations between esds in cell parameters are only used when they are defined by crystal symmetry. An approximate (isotropic) treatment of cell esds is used for estimating esds involving l.s. planes.

### Fractional atomic coordinates and isotropic or equivalent isotropic displacement parameters (Å^2^)

**Table:**

	*x*	*y*	*z*	*U*_iso_*/*U*_eq_	Occ. (<1)
Br1	0.5290 (6)	0.25953 (5)	0.23869 (12)	0.0155 (3)	0.50
Br2	0.2738 (3)	0.27497 (11)	0.07779 (5)	0.0196 (2)	0.50
Br3	0.7682 (3)	0.24565 (11)	0.39355 (5)	0.01777 (18)	0.50
N2	1.1882 (7)	0.2417 (5)	−0.3550 (3)	0.0144 (9)	0.50
N1	0.7232 (7)	0.2399 (4)	−0.10428 (15)	0.0209 (11)	0.50
H1	0.6250	0.2392	−0.0514	0.025*	0.50
C1	0.7724 (9)	0.1050 (3)	−0.1509 (2)	0.0190 (11)	0.50
H1A	0.7000	0.0122	−0.1257	0.023*	0.50
C2	0.9276 (8)	0.1061 (3)	−0.23446 (19)	0.0196 (13)	0.50
H2	0.9612	0.0140	−0.2663	0.024*	0.50
C3	1.0335 (5)	0.2420 (3)	−0.27138 (13)	0.0147 (11)	0.50
C4	0.9844 (9)	0.3768 (3)	−0.2248 (2)	0.0195 (12)	0.50
H4	1.0568	0.4697	−0.2500	0.023*	0.50
C5	0.8292 (9)	0.3757 (3)	−0.1412 (2)	0.0208 (14)	0.50
H5	0.7956	0.4679	−0.1093	0.025*	0.50
C6	1.2376 (13)	0.1015 (6)	−0.4024 (3)	0.0226 (13)	0.50
H6A	1.0314	0.0498	−0.4102	0.034*	0.50
H6B	1.3829	0.0370	−0.3672	0.034*	0.50
H6C	1.3321	0.1220	−0.4620	0.034*	0.50
C7	1.2983 (11)	0.3839 (6)	−0.3936 (4)	0.0223 (14)	0.50
H7A	1.1130	0.4479	−0.4077	0.033*	0.50
H7B	1.4196	0.3638	−0.4493	0.033*	0.50
H7C	1.4366	0.4359	−0.3497	0.033*	0.50

### Atomic displacement parameters (Å^2^)

**Table:**

	*U*^11^	*U*^22^	*U*^33^	*U*^12^	*U*^13^	*U*^23^
Br1	0.0195 (8)	0.01421 (16)	0.0128 (8)	−0.0005 (3)	0.0021 (5)	−0.0007 (2)
Br2	0.0201 (4)	0.0274 (5)	0.0112 (4)	0.0019 (3)	0.0015 (3)	−0.0010 (3)
Br3	0.0210 (4)	0.0207 (4)	0.0116 (4)	−0.0011 (3)	0.0007 (3)	0.0001 (3)
N2	0.021 (2)	0.0110 (19)	0.011 (2)	−0.001 (2)	−0.0034 (17)	0.005 (2)
N1	0.023 (3)	0.032 (3)	0.008 (2)	0.007 (3)	0.0025 (19)	−0.001 (2)
C1	0.019 (3)	0.021 (3)	0.017 (3)	−0.001 (2)	−0.003 (3)	0.002 (2)
C2	0.012 (3)	0.0175 (19)	0.029 (4)	−0.0005 (16)	0.004 (3)	0.003 (2)
C3	0.019 (2)	0.0179 (18)	0.008 (3)	−0.001 (3)	−0.002 (2)	0.0001 (17)
C4	0.020 (2)	0.022 (2)	0.016 (3)	−0.004 (3)	−0.005 (4)	0.0004 (16)
C5	0.019 (3)	0.023 (3)	0.020 (3)	0.001 (2)	−0.001 (3)	0.001 (3)
C6	0.032 (3)	0.019 (2)	0.017 (3)	0.000 (3)	−0.001 (4)	0.004 (2)
C7	0.023 (4)	0.023 (3)	0.020 (3)	0.005 (2)	0.008 (3)	−0.005 (2)

### Geometric parameters (Å, °)

**Table:**

Br1—Br3	2.492 (3)	C2—H2	0.9500
Br1—Br2	2.601 (3)	C3—C4	1.3900
N2—C3	1.390 (5)	C4—C5	1.3900
N2—C6	1.436 (7)	C4—H4	0.9500
N2—C7	1.454 (7)	C5—H5	0.9500
N1—C1	1.3900	C6—H6A	0.9800
N1—C5	1.3900	C6—H6B	0.9800
N1—H1	0.8800	C6—H6C	0.9800
C1—C2	1.3900	C7—H7A	0.9800
C1—H1A	0.9500	C7—H7B	0.9800
C2—C3	1.3900	C7—H7C	0.9800
			
Br3—Br1—Br2	179.41 (8)	C5—C4—H4	120.0
C3—N2—C6	119.9 (4)	C3—C4—H4	120.0
C3—N2—C7	119.4 (4)	C4—C5—N1	120.0
C6—N2—C7	120.7 (4)	C4—C5—H5	120.0
C1—N1—C5	120.0	N1—C5—H5	120.0
C1—N1—H1	120.0	N2—C6—H6A	109.5
C5—N1—H1	120.0	N2—C6—H6B	109.5
N1—C1—C2	120.0	H6A—C6—H6B	109.5
N1—C1—H1A	120.0	N2—C6—H6C	109.5
C2—C1—H1A	120.0	H6A—C6—H6C	109.5
C1—C2—C3	120.0	H6B—C6—H6C	109.5
C1—C2—H2	120.0	N2—C7—H7A	109.5
C3—C2—H2	120.0	N2—C7—H7B	109.5
N2—C3—C4	120.5 (3)	H7A—C7—H7B	109.5
N2—C3—C2	119.5 (3)	N2—C7—H7C	109.5
C4—C3—C2	120.0	H7A—C7—H7C	109.5
C5—C4—C3	120.0	H7B—C7—H7C	109.5
			
C5—N1—C1—C2	0.0	C1—C2—C3—N2	−179.96 (9)
N1—C1—C2—C3	0.0	C1—C2—C3—C4	0.0
C6—N2—C3—C4	179.95 (9)	N2—C3—C4—C5	179.96 (9)
C7—N2—C3—C4	−0.07 (11)	C2—C3—C4—C5	0.0
C6—N2—C3—C2	−0.08 (13)	C3—C4—C5—N1	0.0
C7—N2—C3—C2	179.90 (9)	C1—N1—C5—C4	0.0

### Hydrogen-bond geometry (Å, °)

**Table:**

*D*—H···*A*	*D*—H	H···*A*	*D*···*A*	*D*—H···*A*
N1—H1···Br2	0.88	2.42	3.286 (2)	167
